# Activation of BZW1 by CEBPB in macrophages promotes eIF2α phosphorylation-mediated metabolic reprogramming and endoplasmic reticulum stress in MRL/lpr lupus-prone mice

**DOI:** 10.1186/s11658-023-00494-1

**Published:** 2023-10-12

**Authors:** Huimeng Qi, Zhaoguo Zheng, Qiang Liu

**Affiliations:** 1 Department of General Practice, Fuyang Hospital of Anhui Medical University, Fuyang, 236000 Anhui People’s Republic of China; 2 Department of Nephrology, Guangdong Second Provincial General Hospital, Haizhu District, No. 466, Xingang Zhong, Guangzhou, 510317 Guangdong People’s Republic of China

**Keywords:** CEBPB, BZW1, Glycolysis, Endoplasmic reticulum stress, Lupus nephritis

## Abstract

**Background:**

Lupus nephritis (LN) is associated with significant mortality and morbidity, while effective therapeutics and biomarkers are limited since the pathogenesis is complex. This study investigated the roles of the CEBPB/BZW1/eIF2α axis in metabolic reprogramming and endoplasmic reticulum stress in LN.

**Method:**

The differentially expressed genes in LN were screened using bioinformatics tools. The expression of CEBPB in the renal tissue of patients with LN and its correlation with the levels of creatinine and urinary protein were analyzed. We used adenoviral vectors to construct LN mice with knockdown CEBPB using MRL/lpr lupus-prone mice and analyzed the physiological and autoimmune indices in mice. Chromatin immunoprecipitation quantitative polymerase chain reaction (ChIP–qPCR) and dual-luciferase reporter assays were conducted to explore the regulation of BZW1 by CEBPB, followed by glycolytic flux analysis, glucose uptake, and enzyme-linked immunosorbent assay (ELISA). Finally, the role of eIF2α phosphorylation by BZW1 in bone marrow-derived macrophages (BMDM) was explored using eIF2α phosphorylation and endoplasmic reticulum stress inhibitors.

**Results:**

CEBPB was significantly increased in renal tissues of patients with LN and positively correlated with creatinine and urine protein levels in patients. Downregulation of CEBPB alleviated the autoimmune response and the development of nephritis in LN mice. Transcriptional activation of BZW1 by CEBPB-mediated glucose metabolic reprogramming in macrophages, and upregulation of BZW1 reversed the mitigating effect of CEBPB knockdown on LN. Regulation of eIF2α phosphorylation levels by BZW1 promoted endoplasmic reticulum stress-amplified inflammatory responses in BMDM.

**Conclusion:**

Transcriptional activation of BZW1 by CEBPB promoted phosphorylation of eIF2α to promote macrophage glycolysis and endoplasmic reticulum stress in the development of LN.

## Background

Systemic lupus erythematosus (SLE), a multisystem autoimmune disease, generally affects the kidneys, and lupus nephritis (LN) is the most common cause of kidney injury in patients with SLE [1]. LN is associated with great mortality and morbidity burden, and the development of LN is multifactorial involving genetic susceptibility, environmental triggers, as well as systemic inflammation [2]. Treatment of LN includes immunosuppressive therapy, characteristically with mycophenolate mofetil or cyclophosphamide and with glucocorticoids, and these treatments are proven to be suboptimal [3]. Consequently, exploring the underlying pathogenesis of LN and searching for new targets for LN treatment are urgently needed.

LN is characterized by glomerular deposition of the immune complex (IC), contributing to subendothelial, mesangial, and subepithelial electron-dense deposits, triggering immune cell infiltration and glomerular injury, and macrophages are abundantly present in inflammatory lesions [4]. Furthermore, macrophages undergo a switch to glycolysis in response to IgG IC [5]. Therefore, targeting macrophages may offer potential benefits in LN [6]. We identified here that CCAAT/enhancer-binding protein β (CEBPB) was one of the 28 differentially expressed transcription factors in LN using two Gene Expression Omnibus (GEO) datasets (GSE157293 and GSE153547). Intriguingly, the depletion of CEBPB alleviated the pathological features of LN in MRL/Lpr mice [7]. However, whether it can regulate macrophage glycolysis in LN remains unclear. CEBPB, belonging to the bZIP class of transcription factors that bind specific DNA sequences as homo- and heterodimers, represents a critical regulator of energy and fat metabolism [8]. In addition, by analyzing differentially expressed genes in the two datasets and possible downstream targets of CEBPB in the hTFtarget database, we revealed basic leucine zipper and W2 domain-containing protein 1 (BZW1) as a potential target of CEBPB in LN. BZW1 facilitated glycolysis by potentiating the extent of eukaryotic initiation factor 2 alpha (eIF2α) phosphorylation in pancreatic ductal adenocarcinoma [9]. Phosphorylation of eIF2α on serine 51 is an important strategy in the cell’s armory against stressful insults including viral infection and the accumulation of misfolded proteins [10]. Many conditions that impose stress on cells challenge the folding capacity of the cell and encourage endoplasmic reticulum (ER) stress, and the unfolded protein response intersects with the integrated stress response through the inactivation of eIF2α [11]. Moreover, anti-double-stranded DNA (anti-dsDNA) antibody stimulation significantly enhanced the expression of p-eIF2α in human mesangial cells [12]. Therefore, we postulated that BZW1 activation by CEBPB induced the phosphorylation of eIF2α, thereby enhancing the metabolic reprogramming of macrophages and LN development.

## Materials and methods

### Patients and sample collection

According to the diagnostic criteria for SLE and the level of proteinuria at enrollment (> 0.5 g/24 h), kidney biopsies, and blood and urine samples were collected from untreated patients with LN (*n* = 23) at Fuyang Hospital of Anhui Medical University. Patients aged < 18 years or > 75 years, or patients with hypertension, diabetes mellitus, possible hepatitis B infection, tumors, pregnancy, or other types of kidney-related diseases were not included in this study. Normal control kidney tissue (*n* = 14), blood, and urine samples were obtained from deceased patients who had diseases other than kidney-related diseases at Fuyang Hospital of Anhui Medical University. The relevant information on patients with LN and controls is listed in Table 1. Institutional ethics committee approval was obtained. Written informed consent was acquired from all participants or their relatives.

**Table 1:** Patient demographics for the study

Characteristics	Control (*n* = 14)	Patient with LN (*n* = 23)
Age (years)	35.4 ± 11.2	33.7 ± 10.9
Sex (male/female)	6/8	4/19
BMI (kg/m^2^)	22.17 ± 3.15	22.94 ± 2.86
Blood glucose (mmol/L)	4.76 ± 1.15	4.91 ± 0.84
Serum creatinine (μmol/L)	73.94 ± 21.53	119.53 ± 18.25
24-h urinary protein (g)	0.19 ± 0.04	1.19 ± 0.34
Anti-dsDNA positive (*n*, %)	NA	15 (65.21%)
C3 concentration (g/L)	NA	0.91 ± 0.35

*BMI* body mass index, *anti-dsDNA* anti-double-stranded DNA, *LN* lupus nephritis, *N/A* not applicable

### Mice

Seven-week-old female MRL/lpr mice and wild-type (WT) C57BL/6 J mice were purchased from the Jackson Laboratory (Sacramento, CA, USA) and housed under specific-pathogen-free (SPF) conditions with a 12-h light/dark cycle at 25 ± 1 °C with water and food available. All procedures were conducted following the Guide for the Care and Use of Laboratory Animals published by National Institutes of Health (NIH). All animal study protocols were approved by the animal care and use committee of Anhui Medical University. All mice were acclimatized in a rearing room for 1 week before the start of the experiment.

The mice were divided into six groups: the WT (C57BL/6 J mice), MRL/lpr (MRL/lpr mice), MRL/lpr + short hairpin RNA (sh)-negative control (NC) (injected with adenoviral vectors containing sh-NC), MRL/lpr + sh-CEBPB (injected with adenoviral vectors containing sh-CEBPB), MRL/lpr + sh-CEBPB + overexpression (oe)-NC (injected with adenoviral vectors containing sh-CEBPB + oe-NC), and MRL/lpr + sh-CEBPB + oe-BZW1 groups (injected with adenoviral vectors containing sh-CEBPB + oe-BZW1). A single intravenous (IV) injection of 10^9^ particles of the corresponding plasmid-encapsulated adenovirus vector was administered to the mice during the first week of the experiment, and the packaging and preparation of the adenovirus vector was performed by VectorBuilder (Guangzhou, Guangdong, China). When the mice grew to 24 weeks of age, orbital blood was collected, and 24-h urine was collected in a metabolic cage. After an intraperitoneal injection of 150 mg/kg sodium pentobarbital for euthanasia, the kidney tissue was collected from the mice.

### Biochemical tests

The Creatinine Assay kit (C011-2-1, JianCheng, Nanjing, Jiangsu, China) and Urine Protein Test kit (C035-2-1, JianCheng) were used to analyze serum and urine creatinine and protein levels in mice. The protein-to-creatinine ratio in mice urine was also measured to assess changes in mouse renal function.

### Immunofluorescence

After the euthanasia of mice, kidney tissues were removed, fixed in formaldehyde, and made into paraffin section (4-µm). The sections were deparaffinized in xylene I and xylene II for 15 min each, and rehydrated in ethanol (100%, 95%, 90%, 80%, and 70%) for 5 min, respectively, followed by antigen retrieval using the ethylenediaminetetraacetic acid buffer. The sections were blocked with goat serum for 30 min to remove endogenous non-specific binding sites and cultured with the primary IgG antibody (Abcam, Cambridge, UK) at 4 °C overnight and with fluorescein isothiocyanate (FITC)-coupled goat anti-rabbit secondary antibody (1:1000, ab6717, Abcam) for 1 h at room temperature in the dark. The sections were counter-stained with 4,6-diamidino-2-phenylindole (DAPI), which was applied to visualize the nuclei. The average fluorescence intensity of IgG in the mouse kidney tissues from at least five fields of view was observed under a microscope (200×, Olympus IX71, Olympus Optical Co., Ltd., Tokyo, Japan) after sealing the sections using an antifluorescence-quenching sealing solution.

### Histological analysis

Paraffin-embedded mouse kidney tissues were cut into 3-μm thick paraffin sections. After deparaffinization and rehydration, the histopathological conditions of mouse kidney tissues and the degree of periodic acid–Schiff (PAS)-positive deposition were analyzed using the hematoxylin–eosin (HE) Staining Kit (G1120, Beijing Solarbio Life Sciences Co., Ltd., Beijing, China) and Glycogen PAS Staining Kit (G1281, Solarbio), respectively.

For pathologic scoring of HE staining, uninformed semiquantitative assessment of each mouse kidney tissue was performed by two experienced pathologists according to a previous report [13]. For glomerulonephritis: 0, normal; 1, a mild to moderate increase in cellularity with mesangial proliferation; 2, a moderate increase in cellularity with endocapillary and mesangial proliferation, increased matrix, and/or karyorrhexis; and 3, a marked increase in cellularity with endocapillary proliferation, crescent formation, and/or necrosis, and/or sclerosis.

Uninformed semiquantitative assessment of PAS staining of each mouse kidney tissue was performed by two experienced pathologists according to the relevant scoring criteria [14]. In brief, 0, a healthy condition; 1, mild focal disease; 2, a moderate focal disease; and 3–4, diffuse disease.

### Immunohistochemistry

Paraffin-embedded sections of mouse kidney tissues were deparaffinized and rehydrated, immersed in citrate buffer, then subjected to a heat-mediated antigen retrieval procedure and hydrogen peroxide to remove endogenous peroxidase. After sealing the non-specific binding sites using goat serum, the sections were incubated with CEBPB (1:50, PA5-120,052, Thermo Fisher Scientific Inc., Waltham, MA, USA) and CD68 (1:100, ab283654, Abcam), F4/80 (1:500, 25514, Cell Signaling Technologies, Beverly, MA, USA) at 4 °C overnight and with horseradish peroxidase (HRP)-coupled goat anti-rabbit secondary antibody (1:1000, ab6721, Abcam) for 120 min at room temperature. For this procedure, 3,3′-diaminobenzidine (DAB) was used for color development and hematoxylin for nuclei counter-staining. Finally, the sections were dehydrated and cleared in ethanol and xylene, respectively, and sealed with neutral gum. The number of positive cells was observed under the Olympus IX71 microscope, and the positive rate was calculated.

### Dual-immunofluorescence

To assess the changes in the expression of CEBPB and BZW1 in macrophages of mouse kidney tissues, we used dual-immunofluorescence to label the macrophage marker F4/80 with CEBPB or BZW1, respectively. Mouse kidney tissue sections from each group were incubated in xylene I and xylene II for 15 min each for deparaffinization, followed by incubation in 100%, 95%, 90%, 80%, and 70% ethanol for 5 min for rehydration, respectively. After antigen retrieval of the sections in ethylenediaminetetraacetic acid buffer, the sections were incubated in goat serum for 30 min to remove endogenous non-specific binding sites. The sections were incubated overnight at 4 °C with the following primary antibodies (all from Thermo Fisher): F4/80 (1:100, 14-4801-82), CEBPB (1:100, MA5-32252), or BZW1 (1:200, PA5-35860). This was followed by incubation with goat anti-rat secondary antibody with Alexa Fluor^®^ 488 (1:200, ab150157, Abcam) or goat anti-rabbit secondary antibody with Alexa Fluor^®^ 647 (1:200, ab150079, Abcam), respectively, for 1 h at room temperature. Nuclei were counterstained with DAPI, and images were acquired on the Olympus IX71 microscope.

### Flow cytometry

The peritoneum was removed from freshly isolated mouse kidneys, which were then chopped for homogenization. The homogenized kidney tissue fragments were detached in type I collagenase for 30 min to obtain a single-cell suspension, which was filtered through a 70-μm cell filter. The filtered cell suspension was centrifuged at 300 g for 2 min at 4 °C, and the supernatant was removed. The cells were incubated with erythrocyte lysis buffer (C3702, Beyotime Biotechnology Co., Ltd., Shanghai, China) for 2 min at room temperature to remove erythrocytes, centrifuged at 1000 g for 5 min at 4 °C, washed with phosphate-buffered saline (PBS) containing 1% bovine serum albumin (BSA) and 0.1% sodium azide, and then filtered through a 40-μm cell filter. The final single cell suspension was stained with APC-CD11b (1:50, ab25482, Abcam) and FITC-F4/80 (1:50, ab60343, Abcam) antibodies for 20 min at 4 °C after being blocked by CD16/CD32 antibodies and. FlowJo software was used to analyze the percentage of macrophages in the single-cell suspension of mouse kidney tissues.

### Enzyme-linked immunosorbent assay (ELISA)

LBIS^®^ Anti-dsDNA-Mouse ELISA Kit (631-02699, Amresco, Radnor, PA, USA), Mouse C3 ELISA Kit (ab263884, Abcam), Mouse IL-1 beta/IL-1F2 Quantikine ELISA Kit (MLB00C, R&D Systems, Minneapolis, MN, USA), Mouse IL-6 Quantikine ELISA Kit (M6000B, R&D Systems), and TNF-α (Mouse) ELISA Kit (K1051, BioVision, Inc., Exton, PA, USA) were used.

### Bone marrow-derived macrophages (BMDM) extraction and in vitro stimulation

The femur and tibia were isolated from 6 to 8-week-old C57BL/6 J mice. After cutting the bone, the bone marrow was flushed into Dulbecco’s modified Eagle’s medium (DMEM) containing 2% fetal bovine serum and macrophage colony-stimulating factor. After day 5 of culture, the cells were separated from the culture dish with Tris-buffered saline containing 5 mM EDTA, re-suspended in fresh DMEM, and seeded in 12-well plates.

For in vitro stimulation of BMDM, ovalbumin (Ova), or Ova-IC stimulation was used for 6 h. The BMDM were treated with 5 mM of 2-DG (HY-13966, MedChemExpress, Monmouth Junction, NJ, USA) for 45 min, 10 nmol/μL of 4-PBA (HY-A0281, MedChemExpress) for 1 h, and 1 μM of ISRIB (HY-12495, MedChemExpress) for 4 h to inhibit glycolysis, ER stress, and eIF2α, respectively.

### Transfection

BMDM were seeded into 12-well plates at 3 × 10^5^ cells/well. When the cells grew to 80% confluence, sh-CEBPB, overexpression (oe)-BZW1, and control plasmid (VectorBuilder) were transfected into BMDM using Lipofectamine 2000, respectively. After 24 h, the medium was replaced with a fresh medium, and the transfection efficiency was verified by reverse transcription quantitative polymerase chain reaction (RT–qPCR) after 48 h of further incubation.

### Measurements of metabolic changes

In the Seahorse XFe96 analyzer (Agilent Technologies, Santa Clara, CA, USA), the treated BMDM were seeded in XF96 microtiter plates. Extracellular acidification rate (ECAR) and oxygen consumption rate (OCR) were measured 3–4 times at baseline, and kinetics were recorded for the indicated times after treatment with different concentrations of glucose or inhibitors (oligomycin, FCCP, rotenone, and antimycin A) to calculate ECAR to OCR ratios.

### Glucose uptake assay

Briefly, BMDM were seeded in a 96-well plate and incubated overnight at 37 °C. The cells were starved in serum-free medium for 2 h and incubated with 100 μL Krebs–Ringer phosphate HEPES buffer containing 2% BSA for 40 min to remove endogenous glucose and with 10 μL 2-deoxyglucose (10 mM) for 20 min. The cells were collected with extraction buffer and processed to assay glucose uptake capacity. The glucose uptake level was assessed by measuring the optical density (OD) value in a microplate reader.

### Western blot

Mouse kidney tissues or BMDM were lysed on ice using radioimmunoprecipitation assay (RIPA) lysis buffer, and the supernatant was collected by centrifugation. The protein concentration was examined using the bicinchoninic acid (BCA) method. The protein samples were heated in a water bath, and gel electrophoresis was performed using sodium dodecyl sulfate–polyacrylamide gel electrophoresis (PAGE), followed by transferring the proteins to polyvinylidene difluoride membranes. After blocking with skimmed milk powder for 2 h, the membranes were incubated with primary antibodies at 4 °C overnight. The blots were incubated with the HRP-coupled goat anti-rabbit secondary antibody (1:2000, ab6721, Abcam) for 1 h at room temperature. The signals were visualized using the ECL Western Blotting Substrate (PE0010, Solarbio) and analyzed using Image Pro-Plus 6.0. The primary antibodies were as follows: HK2 (1:1000, 2867, Cell Signaling Technologies), LDHA (1:1000, ab52488, Abcam), BZW1 (1:1000, GTX120426, GeneTex, Inc., Alton Pkwy Irvine, CA, USA), eIF2α (1:1000, 5324, Cell Signaling Technologies), p-eIF2α (1:1000, 3398, Cell Signaling Technologies), CHOP (1:1000, PA5-104528, Thermo Fisher), GRP78 (1:500, PA1-014A, Thermo Fisher), and β-actin (1:200, ab115777, Abcam).

### Reverse transcription–quantitative PCR (RT–qPCR)

Total RNA was extracted from kidney tissues of mice or clinical patients with LN and BMDM using TRIzol reagent and measured for RNA concentration and purity using a spectrophotometer, followed by reverse transcription using TransScript^®^ Reverse Transcriptase [M-MLV, RNaseH-] (AT101-02, Transgene Biotech, Beijing, China). The cDNA was prepared by reaction, followed by qPCR using TransStart^®^ Green qPCR SuperMix (AQ101-01, Transgen). All results were normalized against β-actin expression, and all fold changes were determined using the 2^−ΔΔCT^ method. The primers are listed in Table 2.

**Table 2:** Primers used for RT–qPCR

Gene	Forward sequence (5′–3′)	Reverse sequence (5′–3′)
CEBPB (human)	AGAAGACCGTGGACAAGCACAG	CTCCAGGACCTTGTGCTGCGT
CEBPB (mouse)	CAACCTGGAGACGCAGCACAAG	GCTTGAACAAGTTCCGCAGGGT
BZW1 (human)	TGCAGTAGCTGCAAGTCTTCGG	CTCCGATGGTTTGCTGATTCCG
BZW1 (mouse)	CTGCGAGTCTTCGGAAAGTCAG	GTCTGCTGGTTACGGACATACTC
IL-1β (mouse)	TGGACCTTCCAGGATGAGGACA	GTTCATCTCGGAGCCTGTAGTG
IL-6 (mouse)	TACCACTTCACAAGTCGGAGGC	CTGCAAGTGCATCATCGTTGTTC
TNF-α (mouse)	GGTGCCTATGTCTCAGCCTCTT	GCCATAGAACTGATGAGAGGGAG
β-actin (human)	CACCATTGGCAATGAGCGGTTC	AGGTCTTTGCGGATGTCCACGT
β-actin (mouse)	CATTGCTGACAGGATGCAGAAGG	TGCTGGAAGGTGGACAGTGAGG

*CEBPB* CCAAT/enhancer-binding protein β, *BZW1* basic leucine zipper and W2 domain-containing protein 1, *IL* interleukin, *TNF-α* tumor necrosis factor-alpha

### Chromatin immunoprecipitation (ChIP)–qPCR

Pierce™ Agarose ChIP Kit (26156, Thermo Fisher) was used as per the kit’s instructions to analyze the binding relationship between CEBPB and BZW1. Briefly, BMDM were cross-linked with 1% formaldehyde, and cross-linking was blocked by glycine. The chromatin/DNA complexes were sheared by ultrasonic fragmentation, and the fragmented fractions were incubated with magnetic beads and IP grade CEBPB antibody (1:100, GTX15050, GeneTex) overnight at 4 °C. The elution buffer was used to elute the immunoprecipitated complexes from the magnetic beads before incubation with NaCl at 65 °C for decrosslinking. The cross-linked DNA was purified and extracted for analysis by qPCR after the completion of the crosslinking. qPCR was performed using the following primers: 5′-GCCAGCTCTCAGTTTTGCAT-3′ and 5′-CCGTTCTTTGCATCTCCCAC-3′.

### Dual-luciferase reporter assay

In the University of California Santa Cruz (UCSC) database (http://genome.ucsc.edu/index.html), we obtained the promoter fragment of BZW1 and cloned the fragment of the BZW1 promoter into the pGL3-basic firefly luciferase reporter vector (E1571, Promega Corporation, Madison, WI, USA) using restriction endonuclease. The reporter vector was transfected into BMDM overexpressing CEBPB using Lipofectamine 2000. After 48 h of incubation, luciferase reporter gene activity was measured using the Dual-Luciferase^®^ Reporter Assay System (E1910, Promega).

### Statistics

Results were expressed as mean ± standard deviation (SD). Statistical significance was calculated by performing an unpaired *t*-test, one-way ANOVA, or two-way ANOVA, followed by Tukey's post-test, as appropriate. A *p*-value of < 0.05 was considered statistically significant. GraphPad Software (8.0, San Diego, CA, USA) was used for the statistical analyses. The in vitro experiments were repeated at least three times.

## Results

### CEBPB is significantly upregulated in patients with LN

**Fig. 1:**
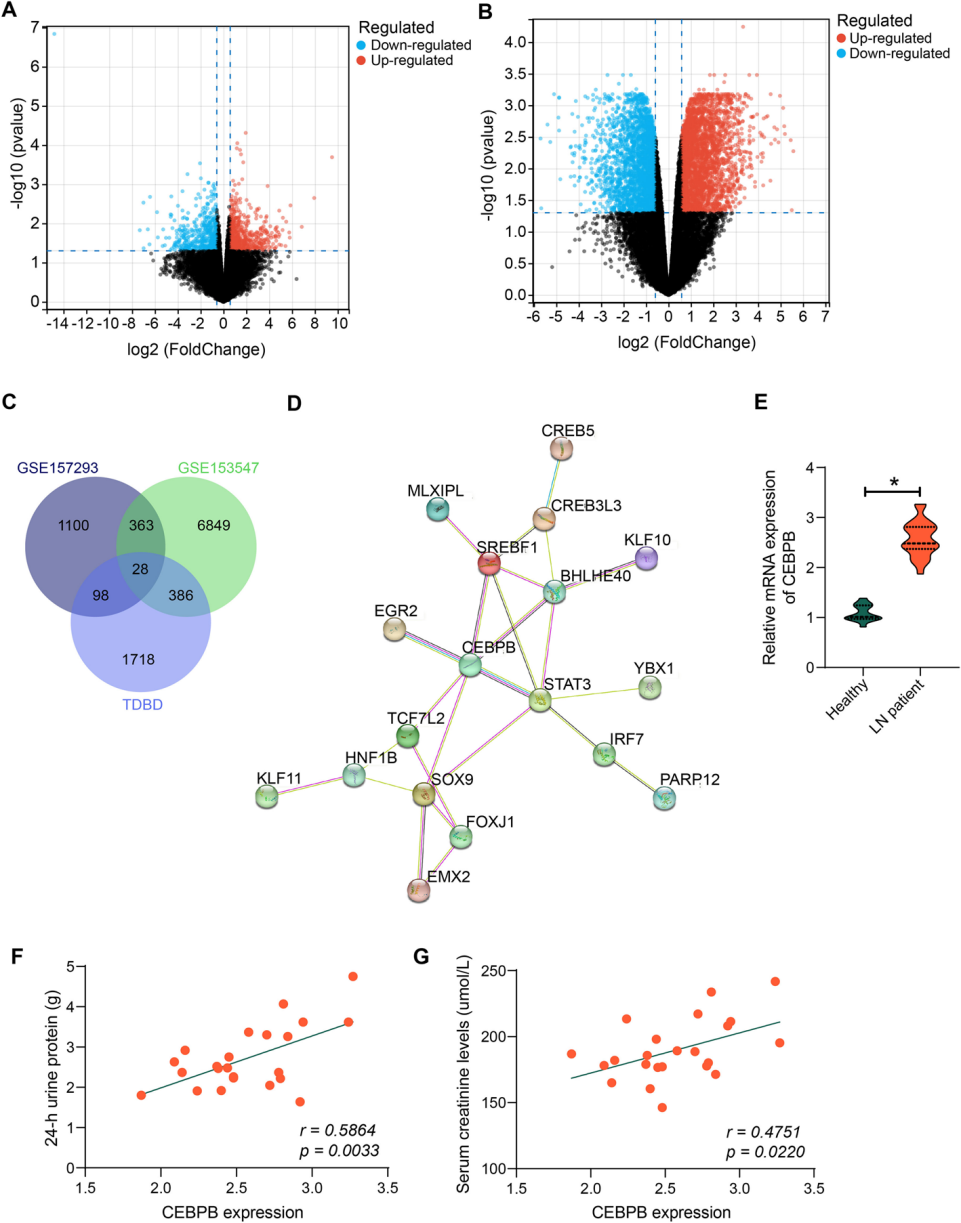
CEBPB is significantly overexpressed in LN. **A** Differentially expressed genes in kidney tissues from patients with LN and healthy kidney tissues (*n* = 3) in the GSE157293. **B** Differentially expressed genes in kidney tissues of LN mice and normal kidney tissues (*n* = 3) in the GSE153547. **C** The intersection of differentially expressed genes in the GSE157293 and GSE153547 datasets. **D** String database analysis of PPI network of 28 differentially expressed genes. **E** RT–qPCR detection of CEBPB expression in kidney tissues of patients with LN (*n* = 23) and healthy kidney tissues (*n* = 14). **F** Correlation of CEBPB expression in kidney tissues of patients with LN and 24-h urine protein content by Pearson’s correlation analysis. **G** Correlation of CEBPB expression in kidney tissues of patients with LN and creatinine levels by Pearson’s correlation analysis. Data are presented as means ± SD (*n* = 8, *p < 0.05). Data were statistically analyzed using unpaired *t*-tests (**E**)

We downloaded the mRNA transcriptome sequencing data from three renal tissues of patients with LN and three healthy renal tissues from patients with cancer (GSE157293) and the expression profile data of kidney tissues from three LN (MRL/lpr) mice and control mice (GSE153547) from the GEO database. Differential analysis was performed using the R package limma (version 3.40.6) to obtain differentially expressed genes in the LN (Fig. 1A, B). Then, we downloaded the list of transcription factors in the Human transcription factor database (TFDB) (http://bioinfo.life.hust.edu.cn/HumanTFDB#!/) and Animal TFDB databases (http://bioinfo.life.hust.edu.cn/AnimalTFDB#!/) and took intersections with the differentially expressed genes in the GSE157293 and GSE153547 datasets (Fig. 1C). A total of 28 intersecting genes were found. Constructing a protein–protein interaction (PPI) network of intersecting genes in the String database, we noted the most widely interacting CEBPB (Fig. 1D).

CEBPB expression was decreased in patients with LN in the GSE157293 dataset (Log_FC_ < 0) and elevated in LN mice in the GSE153547 dataset (Log_FC_ > 0). Due to the small sample size analyzed in the datasets, we subsequently examined its expression in our cohort. RT–qPCR results revealed that CEBPB expression was significantly elevated in the kidney tissues of patients with LN (*n* = 23) relative to healthy kidney tissues (*n* = 14) (Fig. 1E). Moreover, the expression of CEBPB was positively related to the 24-h urine protein and serum creatinine levels in patients (Fig. 1F, G), suggesting that CEBPB expression was significantly elevated in LN and may be positively associated with disease progression in patients.

### Downregulation of CEBPB alleviates LN in mice

**Fig. 2:**
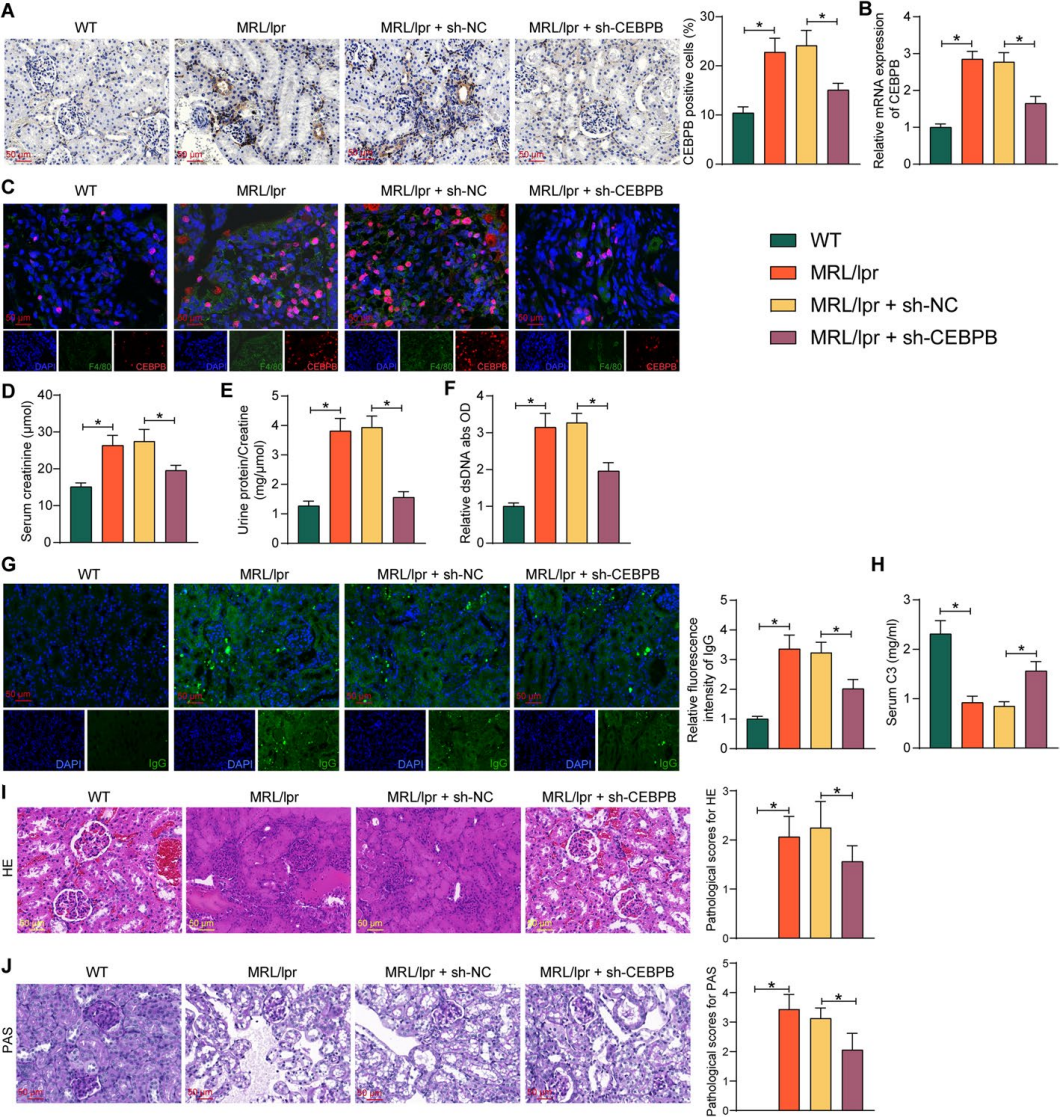
Downregulation of CEBPB alleviates LN in mice. MRL/lpr mice were further treated with sh-NC or sh-CEBPB. **A** Immunohistochemical detection of CEBPB in the kidney tissues of mice. **B** Detection of CEBPB expression in kidney tissues of mice by RT–qPCR. **C** CEBPB expression in macrophages of mouse kidney tissues detected by dual-labeled immunofluorescence. **D** The creatinine levels in the serum of mice. **E** The ratio of urine protein to creatinine in mice. **F** The relative content of dsDNA in the serum of mice. **G** The IgG deposition in the kidney of mice. **H** C3 content in the serum of mice. **I** Histopathological analysis of kidney tissues of mice by HE staining. **J** PAS-positive deposition in the kidney tissues of mice. Data are presented as means ± SD (*n* = 8, *p < 0.05). Data were statistically analyzed using one-way ANOVA

To investigate the role that CEBPB plays in LN, we treated MRL/lpr lupus mice with an adenovirus encapsulating sh-CEBPB or sh-NC. Analysis of CEBPB expression in mouse kidney tissues using immunohistochemistry and RT–qPCR showed a significant increase in CEBPB expression in LN mice and a significant decrease in CEBPB expression in kidney tissues of mice injected with adenoviral vectors containing sh-CEBPB (Fig. 2A, B). Dual-labeled immunofluorescence revealed that the expression of CEBPB was significantly reduced in mouse kidney macrophages after the sh-CEBPB injection (Fig. 2C). Compared with the WT group, MRL/lpr mice showed significantly elevated serum creatinine and urine protein/creatinine levels, while mice with downregulated CRBPB had much lower serum creatinine and urine protein/creatinine levels than the sh-NC group (Fig. 2D, E). Moreover, dsDNA antibody levels were lower in CEBPB-deficient mice than in sh-NC-treated mice (Fig. 2F). In the kidney tissues, IgG deposition was reduced in CEBPB-deficient mice relative to sh-NC-treated mice (Fig. 2G), and conversely, C3 levels were significantly higher in the serum of CEBPB-deficient mice (Fig. 2H). HE staining and PAS staining were conducted using kidney tissues from all groups of mice. It was observed that the kidney tissues of the MRL/lpr mice demonstrated different degrees of glomerular contraction and deformation, glomerular capillary stenosis and occlusion, glomerular thylakoid and endothelial cell proliferation, and interstitial inflammatory cell infiltration. However, the depletion of CEBPB alleviated the diffuse glomerulonephritis and PAS-positive material deposition (Fig. 2I, J).

### CEBPB is associated with macrophage activation in LN mice

**Fig. 3:**
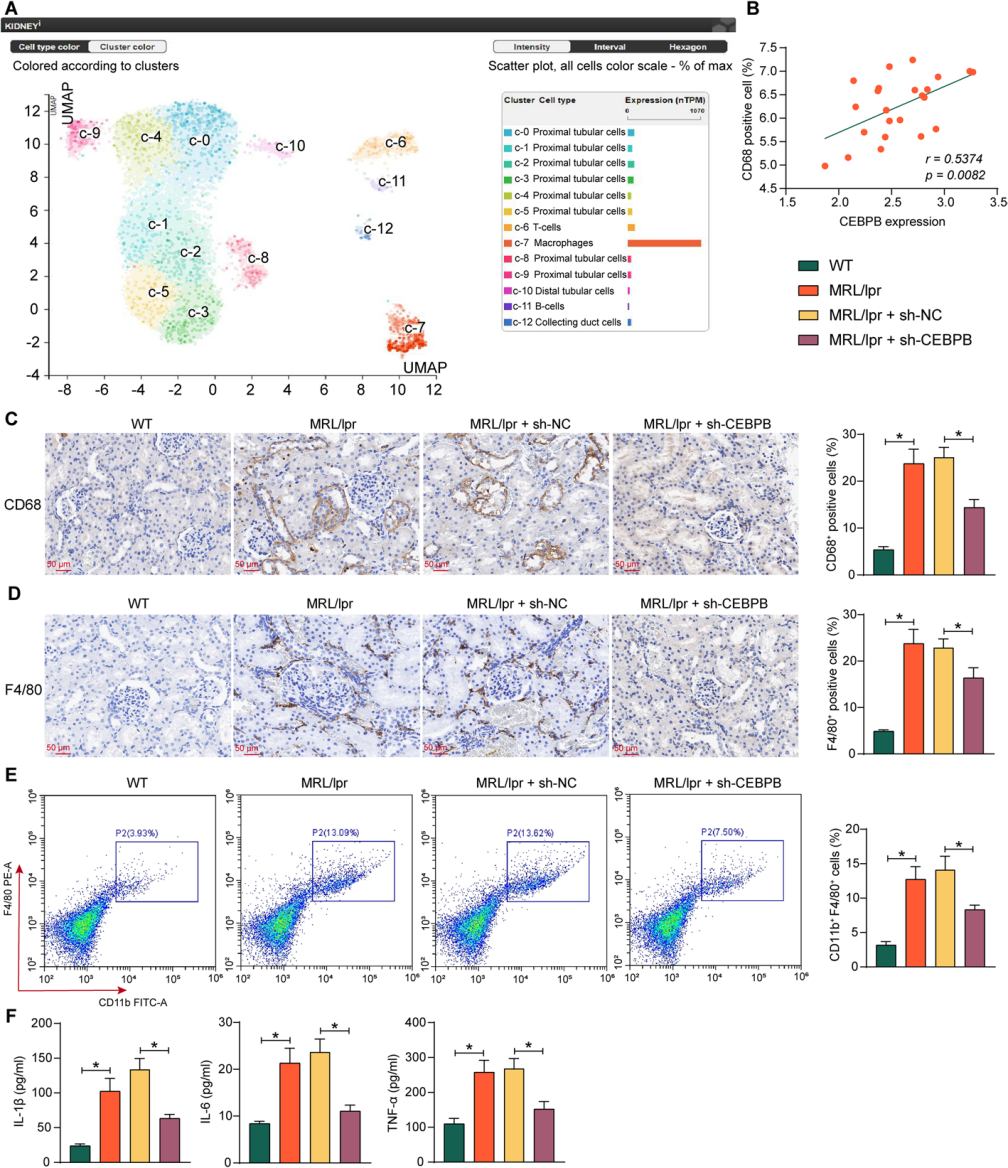
CEBPB is associated with macrophage activation in LN. **A** Analysis of single-cell expression of CEBPB in the kidney using the Human Protein Atlas database. **B** Correlation between CEBPB expression and macrophage infiltration in kidney tissues (*n* = 23) by Pearson’s correlation analysis. **C** Immunohistochemical detection of CD68 in the kidney tissues of LN mice with CEBPB downregulation. **D** Immunohistochemical detection of F4/80 in the kidney tissues of LN mice with CEBPB downregulation. **E** Detection of macrophages in single cell suspensions of mouse kidney tissues by flow cytometry. **F** The IL-1β, IL-6, and TNF-α levels in mouse serum by ELISA. Data are presented as means ± SD (*n* = 8, *p < 0.05). Data were statistically analyzed using one-way ANOVA

In the Human Protein Atlas database (https://www.proteinatlas.org/), CEBPB was found to be predominantly expressed in macrophages in the kidney (Fig. 3A). In the kidney tissues collected from clinical patients with LN, we found a positive correlation between CEBPB expression and the degree of macrophage infiltration (CD68^+^) (Fig. 3B). Immunohistochemical detection of CD68 and F4/80 was conducted to analyze macrophage infiltration in the kidney tissues of LN mice with downregulated CEBPB. Macrophage infiltration was significantly reduced in the kidney tissues of mice injected with the sh-CEBPB adenoviral vector (Fig. 3C, D). Further flow cytometry analysis showed a significant decrease in the percentage of macrophages in the kidneys of mice with CEBPB downregulation (Fig. 3E). Also, the levels of inflammatory response factors IL-1β, IL-6, and TNF-α were significantly reduced in the kidney tissues (Fig. 3F).

### CEBPB induces the transcription of BZW1

**Fig. 4:**
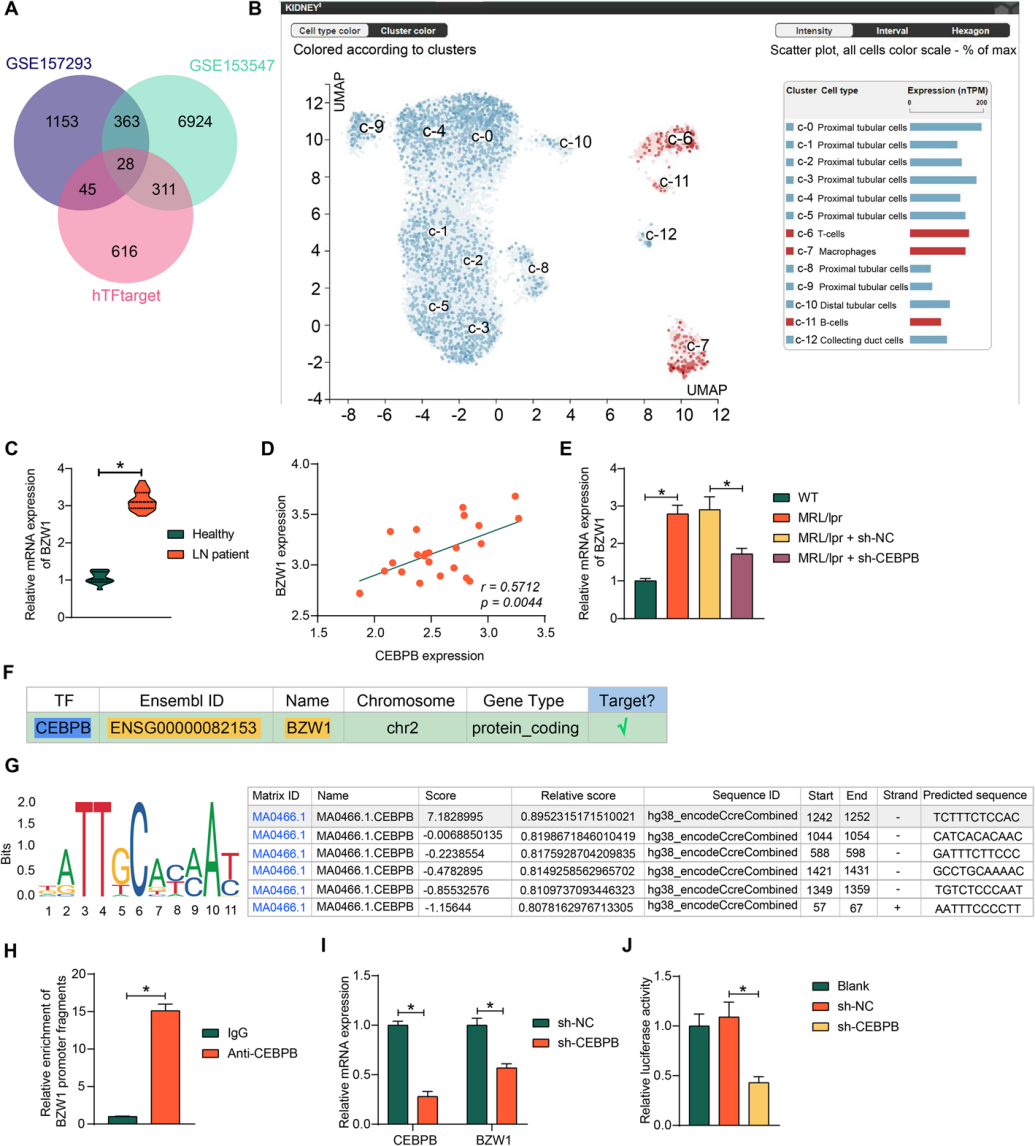
CEBPB induces the transcription of BZW1. **A** Venn diagram of the regulatory downstream targets of CEBPB downloaded from the hTFtarget database and the differentially expressed genes in the GSE157293 and GSE153547 datasets. **B** Analysis of single-cell expression of BZW1 in the kidney by the Human Protein Atlas database. **C** BZW1 expression in kidney tissues of patients with LN (*n* = 23) was analyzed using RT–qPCR. **D** Correlation between BZW1 and CEBPB expression in kidney tissues of patients with LN (*n* = 23). **E** BZW1 expression in kidney tissues of mice was examined using RT–qPCR. **F** BZW1 was predicted as a downstream transcriptional regulatory target of CEBPB using hTFtarget. **G** CEBPB binding sites on BZW1 promoter predicted using the Jaspar database. **H** The binding relation between CEBPB and BZW1 promoter using ChIP–qPCR. **I** CEBPB and BZW1 expression in BMDM with knockdown of CEBPB by RT–qPCR. **J** The transcriptional regulation between CEBPB and BZW1 by dual-luciferase reporter assay. Data are presented as means ± SD and representative of three independent experiments (*n* = 8, *p < 0.05). Data were statistically analyzed using an unpaired *t*-test (**C**, **H**), one-way ANOVA (**E**, **J**), or two-way ANOVA (**I**)

To investigate the downstream regulatory mechanisms of CEBPB in LN, we downloaded the potential downstream transcriptional targets of CEBPB from the hTFtarget database (http://bioinfo.life.hust.edu.cn/hTFtarget#!/) and took intersections of the top 1000 downstream regulatory targets with the differentially expressed genes of LN in the GSE157293 and GSE153547 datasets. A total of 28 differentially expressed genes were obtained (Fig. 4A). It has been reported that macrophages undergo glycolysis after IgG immune complex stimulation, mirroring macrophage metabolic changes in inflamed tissue [5]. Among the 28 intersecting genes, BZW1 has been reported to play crucial roles in glycolysis [9]. The Human Protein Atlas database found BZW1 to be abundantly expressed in macrophages in the kidney (Fig. 4B). However, its effect on macrophages and LN has not been studied yet.

In the GSE157293 dataset, BZW1 showed reduced expression in the patients with LN (Log_FC_ < 0), whereas the GSE153547 dataset showed significantly elevated expression of BZW1 in LN mice (Log_FC_ > 0). Considering the limited sample size of the datasets, BZW1 expression was also evaluated in our cohort. In the kidney tissue of clinical patients with LN, BZW1 expression was significantly elevated and positively correlated with CEBPB expression (Fig. 4C, D). Meanwhile, BZW1 expression was significantly increased in kidney tissues of MRL/lpr mice, while depletion of CEBPB also resulted in BZW1 downregulation (Fig. 4E). The hTFtarget website confirmed that BZW1 is a downstream regulatory target of CEBPB (Fig. 4F). Based on the promoter sequence of BZW1 downloaded from UCSC database, the binding site of CEBPB to the BZW1 promoter was predicted in the Jaspar database (https://jaspar.genereg.net/) (Fig. 4G). In mouse-derived BMDM, the presence of CEBPB binding at the BZW1 promoter was verified using ChIP–qPCR (Fig. 4H). After CEBPB downregulation in BMDM, RT–qPCR showed that sh-CEBPB treatment successfully downregulated the expression of CEBPB, thereby inhibiting BZW1 mRNA expression (Fig. 4G, I). Dual-luciferase reporter assays probing the regulatory relationship between the two confirmed that downregulation of CEBPB led to transcriptional repression of BZW1 (Fig. 4J).

### Activation of BZW1 by CEBPB promotes metabolic reprogramming of macrophages

**Fig. 5:**
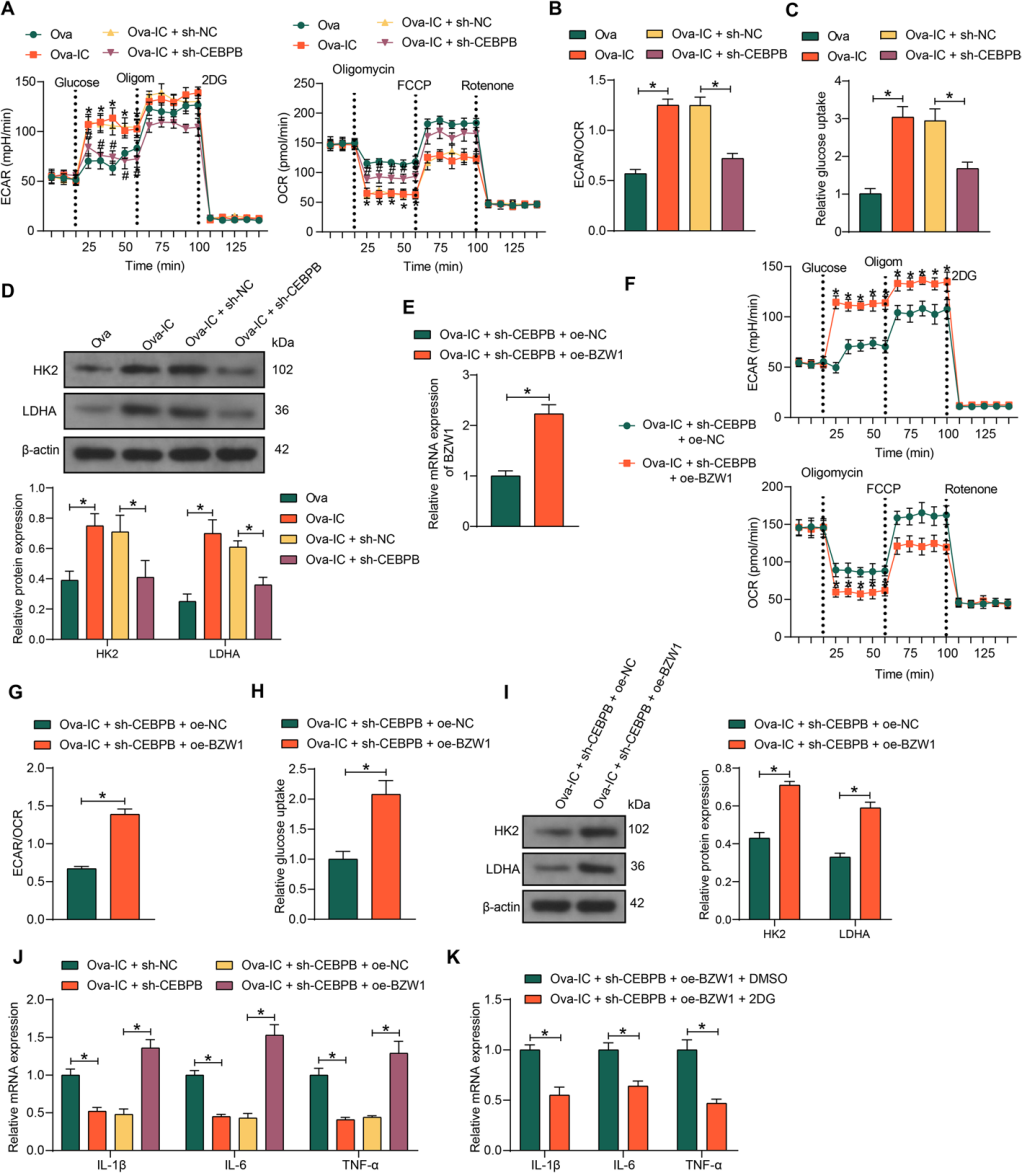
Transcriptional activation of BZW1 by CEBPB promotes metabolic reprogramming of macrophages. BMDM were induced with Ova-IC and further treated with sh-NC or sh-CEBPB. **A** ECAR and OCR traces for BMDM. **B** The ECAR/OCR ratio in BMDM. **C** The glucose uptake capacity of BMDM was measured using a glucose uptake assay. **D** Glycolysis-related protein HK2 and LDHA expression in BMDM were measured using western blot assays. BMDM induced with Ova-IC were further treated with sh-CEBPB + oe-NC or sh-CEBPB + oe-BZW1. **E** BZW1 expression in BMDM after co-transfection. **F** ECAR and OCR traces for BMDM. **G** The ECAR/OCR ratio in BMDM. **H** The glucose uptake capacity of BMDM was measured using a glucose uptake assay. **I** Glycolysis-related protein HK2 and LDHA expression in BMDM were measured using western blot. **J** Expression of IL-1β, IL-6, and TNF-α in BMDM by RT–qPCR. **K** Expression of IL-1β, IL-6, and TNF-α in BMDM in response to 2DG by RT–qPCR. Data are presented as means ± SD and representative of three independent experiments (*p < 0.05). Data were statistically analyzed using an unpaired *t*-test (**E**, **G**, **H**), one-way ANOVA (**B**, **C**), or two-way ANOVA (**A**, **D**, **F**, **I**–**K**)

To investigate the role of CEBPB and BZW1 in macrophage glycolysis in LN, we used an IgG IC model (Ova-IC conditioned with IgG) to stimulate BMDM and knocked down CEBPB in the cells to assess their glucose metabolism levels. Ova-IC-stimulated BMDM showed a significant promotion in ECAR levels and a decline in OCR levels (Fig. 5A), leading to increased ECAR/OCR ratio (Fig. 5B). Furthermore, enhanced glucose uptake (Fig. 5C) and increased expression of HK2 and LDHA were observed in BMDM induced with Ova-IC (Fig. 5D). By contrast, downregulation of CEBPB in the BMDM blocked the switch to glycolysis.

Then, we further overexpressed BZW1 in BMDM and verified the success of overexpression using RT–qPCR (Fig. 5E). Interestingly, we found that cellular glycolysis levels partially recovered after BZW1 upregulation (Fig. 5F–I), with elevated ECAR, downregulated OCR levels, increased ECAR/OCR ratio, and significantly promoted levels of glucose uptake and glycolysis-related proteins. Downregulation of CEBPB expression inhibited IL-1β, IL-6, and TNF-α expression, while ectopic expression of BZW1 restored IL-1β, IL-6, and TNF-α expression (Fig. 5J). When the BMDM overexpressing BZW1 were further treated with the glycolysis inhibitor 2DG, the levels of IL-1β, IL-6, and TNF-α in the cells were significantly reduced (Fig. 5K), indicating that the inflammatory response of macrophages by CEBPB/BZW1 is related to the metabolic shift of glycolysis.

### Overexpression of BZW1 mitigates the alleviating effects of sh-CEBPB in mice with LN

**Fig. 6:**
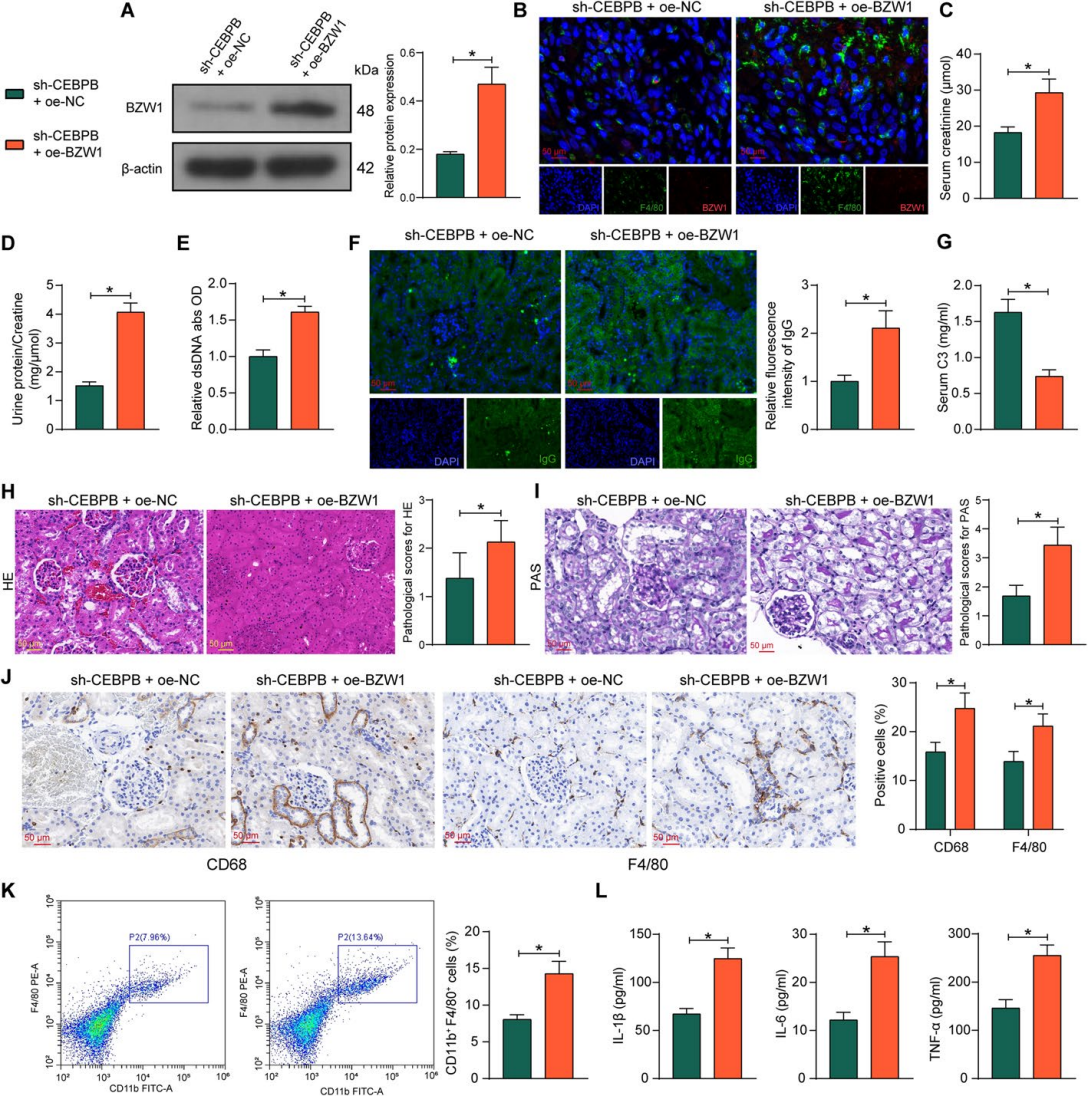
Overexpression of BZW1 overturns the mitigating effects of sh-CEBPB on LN in mice. MRL/lpr mice were further treated with sh-CEBPB + oe-NC or sh-CEBPB + oe-BZW1. **A** The protein expression of BZW1 in mouse kidney tissues was measured using western blot assays. **B** Expression of BZW1 in mouse kidney macrophages detected by dual-label immunofluorescence. **C** The creatinine levels in the serum of mice. **D** The ratio of urine protein to creatinine in mice. **E** The relative content of dsDNA in the serum of mice. **F** The IgG deposition in the kidney of mice. **G** C3 content in the serum of mice. **H** Histopathological analysis of kidney tissues of mice by HE staining. **I** PAS-positive deposition in the kidney tissues of mice. **J** Immunohistochemical detection of CD68 and F4/80 in the kidney tissues of mice. **K** CD11b^+^CD68^+^ macrophages in single cell suspensions of mouse kidney tissues analyzed by flow cytometry. **L** Expression of IL-1β, IL-6, and TNF-α in kidney tissues of mice was measured using ELISA. Data are presented as means ± SD (*n* = 8, *p < 0.05). Data were statistically analyzed using an unpaired *t*-test (**A**, **C**–**I**, **K**–**L**) or two-way ANOVA (**J**)

We overexpressed BZW1 in LN mice with sh-CEBPB (named sh-CEBPB + oe-BZW1 and sh-CEBPB + oe-NC as control). Western blot analysis showed that the expression of BZW1 was significantly elevated in mouse kidney tissues after sh-CEBPB + oe-BZW1 treatment (Fig. 6A), while dual-labeled immunofluorescence also confirmed that the expression of BZW1 was enhanced in mouse kidney macrophages (Fig. 6B). Analysis of the indicators in LN mice showed that BZW1 upregulation led to an increase in creatinine levels in LN mice (Fig. 6C) and a higher urine protein/creatinine ratio than in the sh-CEBPB + oe-NC group (Fig. 6D). Correspondingly, the serum levels of dsDNA antibody and IgG deposition in the kidney was also higher (Fig. 6E, F) and serum C3 level was lower in mice with sh-CEBPB and oe-BZW1 treatment (Fig. 6G), indicating that the upregulation of BZW1 reversed the mitigating effect of sh-CEBPB on the autoimmune response to LN.

Histological staining showed that the upregulation of BZW1 exacerbated deposition of PAS-positive material (Fig. 6H, I), significantly deteriorated macrophage infiltration in kidney tissues (Fig. 6J), increased the number of macrophages in the single cell suspension of the kidney (Fig. 6K), and a corresponding rise in proinflammatory factors (Fig. 6L).

### BZW1 promotes the phosphorylation of eIF2α to enhance macrophage glycolysis and ER stress

**Fig. 7:**
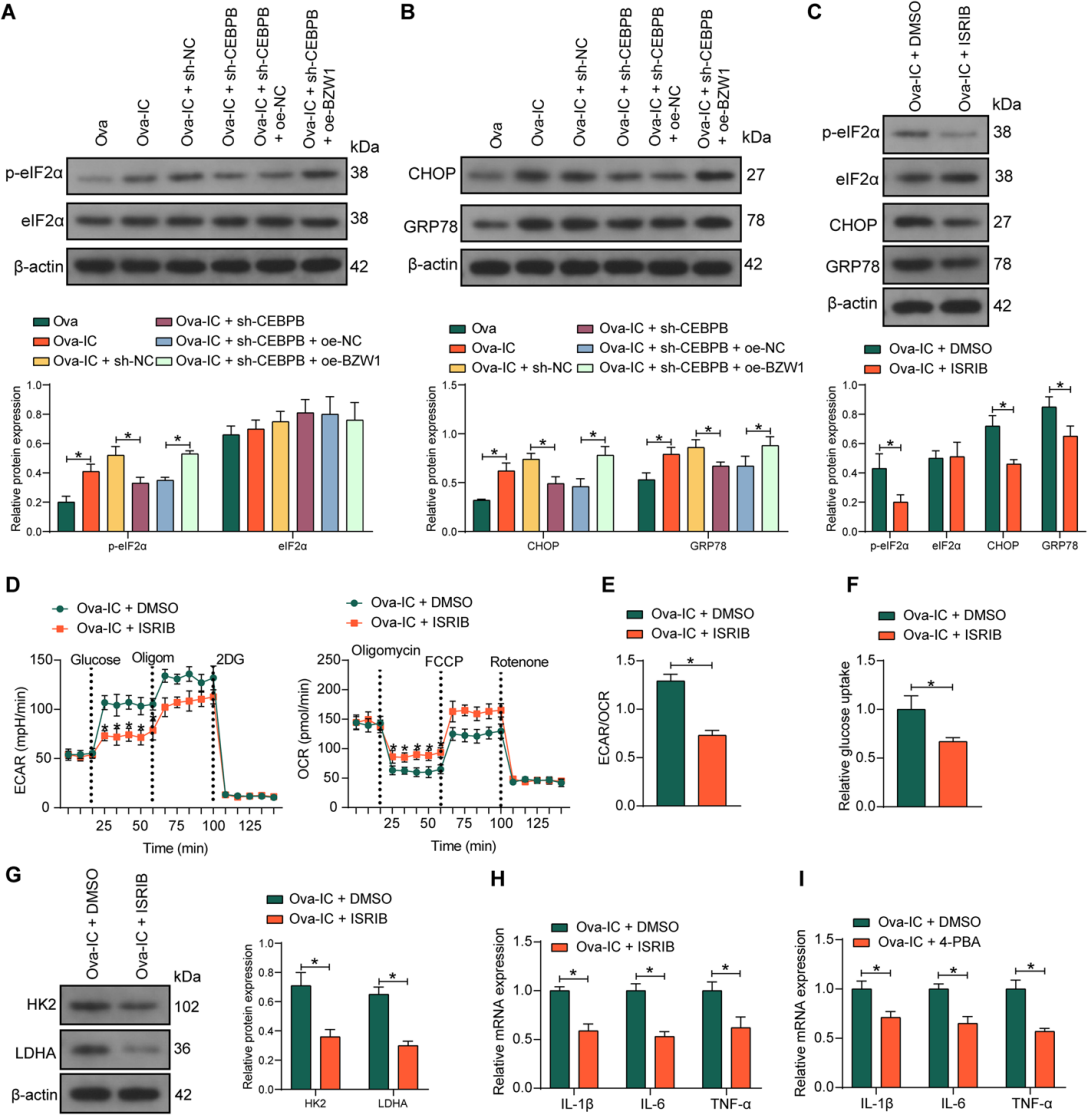
BZW1 phosphorylates eIF2α to promote macrophage glycolysis and ER stress. BMDM induced with Ova-IC were further treated with sh-CEBPB + oe-NC or sh-CEBPB + oe-BZW1. **A** The total protein level and phosphorylation levels of eIF2α in Ova-IC-treated BMDM. **B** The expression of ER stress-related proteins CHOP and GRP78 in Ova-IC-treated BMDM. **C** The protein expression of p-eIF2α, eIF2α, CHOP, and GRP78 expression in BMDM. **D** ECAR and OCR traces for BMDM. **E** The ECAR/OCR ratio in BMDM. **F** The glucose uptake capacity of BMDM was measured using a glucose uptake assay. **G** Glycolysis-related protein HK2 and LDHA expression in BMDM were measured using western blot. **H** Expression of IL-1β, IL-6, and TNF-α in BMDM by RT–qPCR. **I** Expression of IL-1β, IL-6, and TNF-α in BMDM in response to 4-PBA by RT–qPCR. Data are presented as means ± SD and representative of three independent experiments (*p < 0.05). Data were statistically analyzed using an unpaired *t*-test (**E**, **F**) or two-way ANOVA (**A**–**D**, **G**–**I**)

We evaluated the phosphorylation of eIF2α in BMDM and found that Ova-IC promoted eIF2α phosphorylation, which was inhibited by the downregulation of CEBPB. Furthermore, eIF2α phosphorylation levels increased after overexpression of BZW1 even in the presence of sh-CEBPB (Fig. 7A). There was an increase in the expression of the ER stress-inducible chaperones GRP78 and CHOP in isolated glomeruli after LN [12]. We examined the expression of CHOP and GRP78 in BMDM after co-transfection. Ova-IC promoted ER stress in BMDM, while downregulation of CEBPB decreased the protein expression of CHOP and GRP78. As expected, BZW1 promoted ER stress in the presence of sh-CEBPB (Fig. 7B).

We treated BMDM with the phosphorylated eIF2α inhibitor ISRIB, which led to inhibited protein expression of CHOP and GRP78 in the cells and the phosphorylation level of eIF2α (Fig. 7C). Also, inhibition of eIF2α phosphorylation resulted in a significant decrease in cellular glycolysis levels (Fig. 7D–G) and a decrease in proinflammatory factor levels in cells (Fig. 7H). In addition, we treated BMDM with the ER stress inhibitor 4-PBA and found that the blockade of ER stress suppressed the expression of proinflammatory factors in BMDM (Fig.7I). Therefore, it was suggested that ER stress is related to inflammatory signaling in BMDM.

## Discussion

LN is a major reason of morbidity and mortality in lupus, and new approaches for improving patient prognosis and monitoring treatment efficacy are needed [15]. A recent clinical study proposed that among the 76 proliferative LN cases, the number of CD68 macrophage infiltrates was positively correlated with serum creatinine level and tubulointerstitial inflammation [16]. This present study demonstrated that CEBPB was highly expressed in the kidneys of patients and mice with LN. However, sh-CEBPB treatment reduced the metabolic reprogramming in macrophages, thereby ameliorating LN in mice. Furthermore, using an in vitro BMDM model, our study revealed that CEBPB activated the expression of BZW1 to potentiate the phosphorylation of eIF2α, thus enhancing ER stress. These results suggest that inhibition of CEBPB represents a strategy for treating LN.

CEBPB has been reported to promote microRNA-16 expression, resulting in aggravated ischemia/reperfusion-induced acute kidney injury [17]. In the present study, we observed the suppressing effects of sh-CEBPB on serum creatinine, urine protein/creatinine, and relative dsDNA levels. Total glucosides of peony have been revealed to increase the frequency of splenic and peritoneal F4/80^+^CD11b^+^CD206^+^ M2-like macrophages, thereby ameliorating pristane-induced LN [18]. Furthermore, CEBPA was identified as one of the key genes differentially expressed in macrophage response to *Staphylococcus aureus* exposure [19]. More relevantly, Zahid et al. observed that CEBPB knockdown reduced inflammation, ER stress, and apoptosis, and promoted autophagy in oxLDL‑treated RAW264.7 macrophages [20]. These findings highlighted the regulatory role of CEBPB in macrophages. As expected, the positive correlation between CEBPB and macrophage infiltration has been confirmed here. Macrophages can be simplified into two extremes: proinflammatory (M1) and antiinflammatory/pro-resolving (M2), and M1 macrophages rely mainly on glycolysis for metabolism [21]. We further verified the alleviating effects of sh-CEBPB on the activation of macrophages and inflammatory responses in mice with LN. Combined with several bioinformatics tools, BZW1 was identified as one of the targets of CEBPB in LN. BZW1 is a conserved factor for transcriptional control of the histone H4 gene at the G1/S transition [22]. The function of BZW1 in LN has not yet been well established, which makes it our gene of interest.

To substantiate the binding relation between BZW1 and CEBPB and to characterize the function of BZW1 in LN, we extracted BMDM from mice and induced them with Ova-IC for in vitro study. Increased ECAR but not OCR was observed in IL-23-stimulated primary renal tubular epithelial cells [23]. The metabolic reprogramming allows the macrophages to the microenvironment alterations and to fulfill their highly energy-demanding proinflammatory and antimicrobial functions [24]. Accordingly, we observed that depletion of CEBPB contributed to impaired glycolysis in BMDM stimulated with Ova-IC, as evidenced by lowered ECAR/OCR, glucose intake, and glycolysis-related protein expression. Since BZW1 is possibly activated by CEBPB in LN, we overexpressed BZW1 in BMDM with the knockdown of CEBPB to observe the glycolysis changes. Restoration of BZW1 significantly enhanced the glycolysis of BMDM and the ensuing inflammatory response. These in vitro findings were reproduced in vivo in LN mice.

Kepp et al. reported that eIF2α phosphorylation was required for the preapoptotic exposure of the ER chaperone calreticulin on the cell surface, which is a major determinant of immunogenic cell death [25]. Moreover, eIF2α phosphorylation has been associated with the regulation by BZW1 [9]. Here, we observed the restoration of eIF2α phosphorylation in response to the overexpression of BZW1 even in the presence of sh-CEBPB. Lipopolysaccharide-induced ER stress in human periodontal ligament cells activated the expression of CEBPB, which is involved in the regulation of ER stress in human periodontal ligament cells in response to lipopolysaccharide stimuli [26]. After the application of the eIF2α phosphorylation inhibitor, we observed here that the glycolysis switch and ER stress induced by BZW1 were rescued. It is generally acknowledged that ER stress and inflammation are two mechanisms that allow tissue and cells to adapt to infections or stress-induced situations, and ER stress inhibitor 4-PBA has been revealed to significantly decrease the levels of anti-dsDNA antibodies and serum TNF-α, as well as the glomerulonephritis score in an experimental murine lupus model [27]. Therefore, the novelty of this study lies in the association between eIF2α phosphorylation caused by CEBPB-induced BZW1 transcriptional activation and ER stress-related inflammation.

In active proliferative LN, initial treatment with mycophenolate mofetil or low-dose intravenous cyclophosphamide, both combined with glucocorticoids (pulses of intravenous methylprednisolone, then oral prednisone 0.3–0.5 mg/kg/day), is recommended [28]. Glucocorticoids and synthetic glucocorticoids have two mechanisms of action: the genomic and non-genomic mechanisms, and the former mainly involve a decreased transcription of genes encoding inflammatory cytokines and an increased transcription of antiinflammatory genes [29]. Since glucocorticoids are related to ER stress under many conditions, the impact of these medications on the axis we studied awaits additional studies. In addition, further specific knockout of CEBPB in the macrophages is warranted to support our conclusion.

## Conclusions

**Fig. 8:**
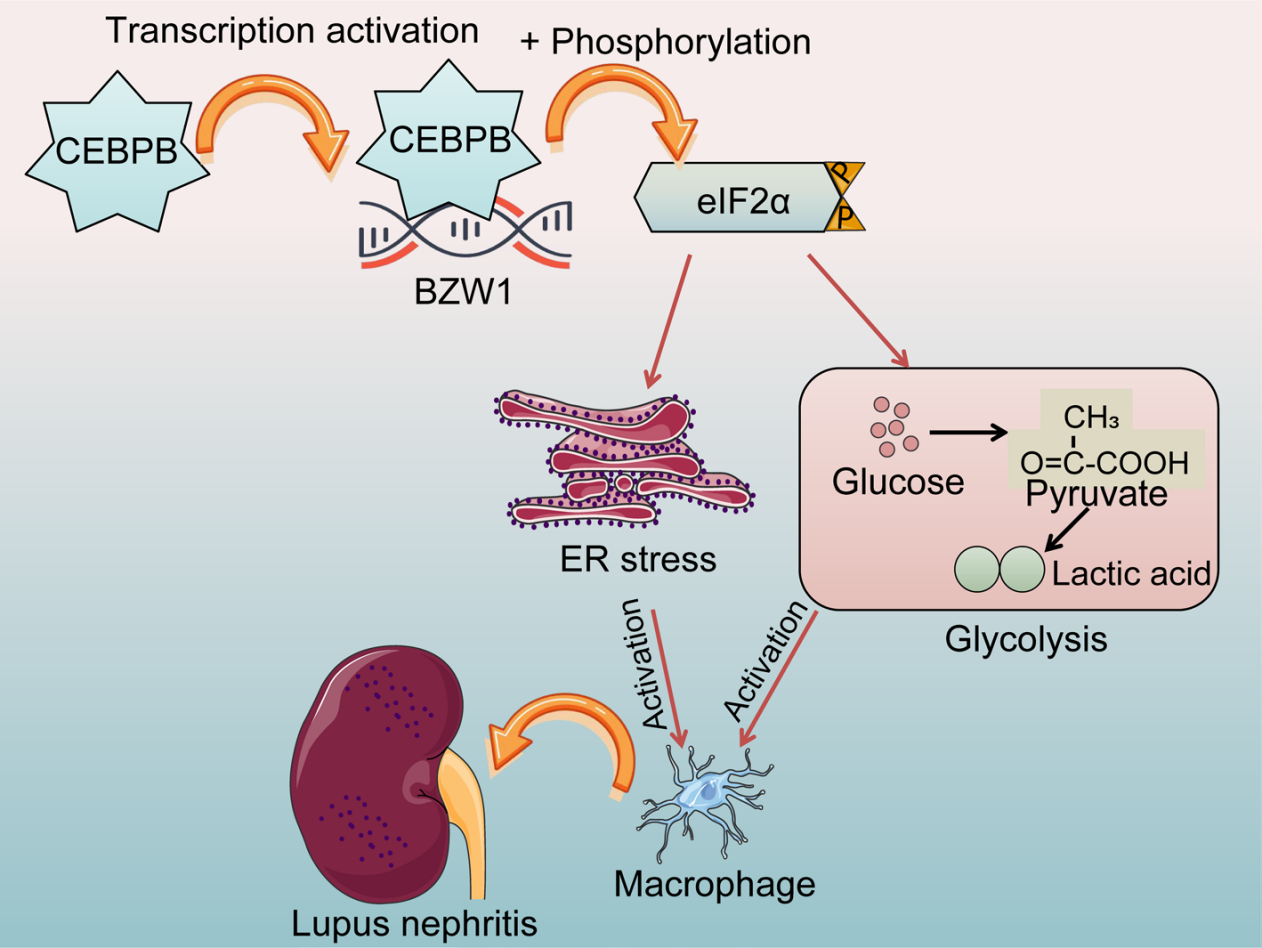
Schematic illustration. CEBPB activation of BZW1 induces phosphorylation of eIF2α to promote macrophage glycolysis and ER stress in the development of LN

In summary, with a combination of a mouse model and cell model of LN, we demonstrate the essential role of the CEBPB/BZW1/eIF2α axis in metabolic reprogramming and ER stress during LN (Fig. 8). This study provides mechanistic insights into LN and thus helps us develop better strategies to treat LN.

## Data Availability

All data during the study are available from the corresponding author by request.

## Abbreviations

LN: Lupus nephritis
CEBPB: CCAAT/enhancer-binding protein β
BZW1: Basic leucine zipper and W2 domain-containing protein 1
eIF2α: Eukaryotic initiation factor 2 alpha
ER: Endoplasmic reticulum
WT: Wild-type
FITC: Fluorescein isothiocyanate
PAS: Periodic acid-Schiff
HE: Hematoxylin–eosin
HRP: Horseradish peroxidase
BSA: Bovine serum albumin
DMEM: Dulbecco’s modified Eagle’s medium
Ova: Ovalbumin
IC: Immune complex
ECAR: Extracellular acidification rate
OCR: Oxygen consumption rate
OD: Optical density
ChIP: Chromatin immunoprecipitation
